# Dynamic Expression of Genes Encoding Ubiquitin Conjugating Enzymes (E2s) During Neuronal Differentiation and Maturation: Implications for Neurodevelopmental Disorders and Neurodegenerative Diseases

**DOI:** 10.3390/genes15111381

**Published:** 2024-10-26

**Authors:** Agathe Paubel, Sylviane Marouillat, Audrey Dangoumau, Cindy Maurel, Shanez Haouari, Hélène Blasco, Philippe Corcia, Frédéric Laumonnier, Christian R. Andres, Patrick Vourc’h

**Affiliations:** 1 Université de Tours, INSERM, Imaging Brain & Neuropsychiatry iBraiN U1253, 37044 Tours, France; dr.agathepaubel@gmail.com (A.P.); audrey.dangoumau@univ-tours.fr (A.D.); cindy.maurel@mq.edu.au (C.M.); shanez.ha@gmail.com (S.H.); helene.blasco@univ-tours.fr (H.B.); philippe.corcia@univ-tours.fr (P.C.); frederic.laumonnier@univ-tours.fr (F.L.); christian.andres@univ-tours.fr (C.R.A.); 2 Service de Biochimie et Biologie Moléculaire, CHU de Tours, 37032 Tours, France; 3 Service de Neurologie, Centre SLA, CHU de Tours, 37032 Tours, France

**Keywords:** post-translational modifications, ubiquitin, SUMO, intellectual disability, Alzheimer, Parkinson, amyotrophic lateral sclerosis

## Abstract

**Background:** The ubiquitination process plays a crucial role in neuronal differentiation and function. Numerous studies have focused on the expression and functions of E3 ligases during these different stages, far fewer on E2 conjugating enzymes. In mice, as in humans, these E2s belong to 17 conjugating enzyme families. **Objectives:** We analyzed by real-time PCR the expression dynamics of all known E2 genes during an in vitro differentiation of mouse hippocampal neuronal cultures, and after, we analyzed their stimulation with N-methyl-D-aspartate (NMDA). **Results:** We found that 36 of the 38 E2 genes were expressed in hippocampal neurons. Many were up-regulated during neuritogenesis and/or synaptogenesis stages, such as *Ube2h*, *Ube2b*, and *Aktip*. Rapid and delayed responses to NMDA stimulation were associated with the increased expression of several E2 genes, such as *Ube2i*, the SUMO-conjugating E2 enzyme. We also observed similar expression profiles within the same E2 gene family, consistent with the presence of similar transcription factor binding sites in their respective promoter sequences. **Conclusions:** Our study indicates that specific expression profiles of E2 genes are correlated with changes in neuronal differentiation and activity. A better understanding of the regulation and function of E2s is needed to better understand the role played by the ubiquitination process in physiological mechanisms and pathophysiological alterations involved in neurodevelopmental or neurodegenerative diseases.

## 1. Introduction

The ubiquitin (Ub) and ubiquitin-like (Ubl) pathways are evolutionarily highly conserved post-translational mechanisms that enable the covalent tagging of cellular proteins by Ub or Ubl [1]. The ubiquitination process requires three types of enzymes. First, E1 activating enzymes form a thioester bond with Ub in an ATP-dependent reaction. Next, E2 conjugating enzymes transfer Ub or Ubl to target proteins either directly or indirectly using E3 ligases [2]. E3 RING accepts Ub from E2 before transferring them to target substrates. E3 HECT acts as a scaffold to position substrates close to E2, facilitating the direct transfer of Ub from E2 to the target protein. The specificity of the Ub or Ubl attachment to target proteins is mainly dictated by the E2 and E3 enzymes.

Ubiquitination is a key mechanism in many cellular processes in neurons, participating in protein turnover with the proteasome, membrane protein trafficking, or internalization, for example. There are several ubiquitin-like (Ubl) proteins [1], such as SUMO, NEDD8, and ISG15. SUMO1-4 (small ubiquitin modifiers 1 to 4) and ubiquitin share approximately 20% of amino acids [3]. Various studies indicated important roles for the SUMO pathway, particularly in neurons during differentiation, synaptic formation, and physiology [4,5] Another Ubl protein, NEDD8 (neural precursor cell expressed; developmentally down-regulated 8), shares 60% amino acid sequence identity with Ub, participating in the neddylation of cullin-containing E3 complexes [6]. Protein neddylation does not lead to the degradation of proteins but modifies their localization or function, which is a necessary molecular mechanism involved in synapse formation [7]. Finally, ISG15 (interferon-stimulated gene 15) has 30% identity with ubiquitin. Protein ISGylation is involved in increasing or decreasing protein concentrations by competing with or promoting degradation via the ubiquitin–proteasome system (UPS) or the lysosome-associated pathway [8,9,10].

Numerous PTMs, such as ubiquitination, affecting proteins during or after their synthesis are directly involved in the pathophysiology of many diseases affecting the central nervous system (CNS). These modifications under pathophysiological conditions correspond to what we have recently defined as post-translational variants (PTVs) [11]. Defects or dysfunctions of the Ub and Ubl pathways have been reported in a variety of human diseases, including neurodevelopmental disorders (NDDs) and neurodegenerative diseases (NDs) [12,13,14]. For example, cognitive deficits have been observed in the presence of pathogenic variants in E2 and E3 encoding genes, as seen in Angelman syndrome (*UBE3A*) and intellectual disability (*UBE2A*, *UBCl1*, *CUL4B*, *HUWE1*, *BRWD3*) [15,16,17,18,19,20,21]. Studies also support the role of the E2 and E3 genes in autism spectrum disorder, another NDD [22,23,24]. Regarding NDs, Ub- and SUMO-positive cellular and nuclear inclusions are observed in NDs such as Alzheimer’s disease, Parkinson’s disease, or amyotrophic lateral sclerosis (ALS) [25,26,27]. Mutations in Ub pathway genes such as *FUS* and *PARKIN* have been reported in ALS and Parkinson’s disease, respectively [28,29]. It is also important to note that these genes encoding E2s and E3s are targets or tools of growing interest for the development of new therapeutic strategies for diseases of the CNS.

Functional studies have indeed implicated several genes of the Ub pathway in key processes of neuronal function, such as the regulation of neurite development, synapse formation and elimination, and pre- and post-synaptic function [30,31,32,33,34,35]. The vast majority of studies to date on the involvement of the Ub pathway in neuronal physiology and pathophysiology has focused on E3s, while little attention has been paid to E2s. However, the few studies performed on these E2s demonstrate their great interest [36,37]. The origin of eukaryotic E2s can be traced back to the biflagellate protist *Guillardia theta*, approximately 2500 million years ago [38]. We have reported the presence of 17 E2 protein families, with 37 E2 genes in the human genome [39]. More specifically, 29 genes encode enzymes dedicated specifically to Ub conjugation, 3 enzymes to both Ub and ISG15 conjugation (mainly *UBE2L6* but also *UBE2E1*, *UBE2E2*), 1 enzyme to SUMO conjugation (*UBE2I*), and 2 enzymes to NEDD8 conjugation *(UBE2M*, *UBE2F*) [40]. The functions of *UBE2NL* are still unknown according to the literature.

The aim of the study was to investigate E2 expression in neurons in order to better understand their roles. Having identified all the genes encoding E2 enzymes in the mouse genome based on a previous phylogenetic study of several species [39], an analysis of their expression in primary cultures of mouse hippocampal neurons was performed using the robust RT-qPCR method. Hippocampal neurons are a widely used model for studies on neuronal differentiation, maturation, and activity as well as studies on pathophysiological processes in neurodevelopmental and neurodegenerative diseases. Finally, an in silico analysis of E2 gene promoters provided information on the possible regulation of their expression in neurons.

## 2. Materials and Methods

### 2.1. Primary Cultures of Hippocampal Neurons

Experiments on mice were performed according to protocols approved by the Animal Experimentation Ethic Committee (CEEA) Val de Loire (France). Primary cultures were prepared from C57BL/6J mice at gestation day E17.5 according to the protocol of Laumonnier and collaborators [41]. Briefly, hippocampi were dissected from embryos under binocular magnifier using scissors and triturated in phosphate-buffered saline (PBS) before incubation with papain (10 U/mL) (Worthington, Lakewood, CA, USA) for 22 min at 37 °C. Cells were resuspended in Dulbecco’s modified Eagle’s medium/F12 with 10% fetal bovine serum (Invitrogen, Carlsbad, CA, USA), then centrifuged at 370× *g* for 3.5 min. The pellet was resuspended in Neurobasal/B27/0.5 mM glutamine (Invitrogen). Dissociated cells were plated on glass coverslips coated with poly-D-lysine (50 µg/mL; Sigma, Saint Louis, MO, USA) and laminin (5 µg/mL; Sigma) at a density of 400 cells per mm^2^ and placed in an incubator thermoscientific Heracell 150i (Thermofisher, Waltham, MA, USA)at 37 °C and 5% CO_2_. Half the medium was changed every three days. Mature neurons were stimulated with N-methyl-D-aspartate (NMDA) on day 17. Mature neurons were washed with Tris-buffered control salt solution containing 25 mM Tris-HCl (pH 7.4), 15 mM D-glucose, 5.4 mM KCl, 120 mM NaCl, and 1.8 mM CaCl2. Next, NMDA (50 µM, Sigma) and glycine (10 µM, Sigma) in control salt solution were added to the culture for 5 min. The cells were washed and replaced with normal medium and placed in the incubator.

### 2.2. Immunocytochemistry

Cells were fixed with cold 4% paraformaldehyde for 30 min (days 4, 8, and 17). After fixation, preparations were incubated for 30 min with blocking solution (10% normal donkey serum, 0.2% Triton X100) and for 1 h with rabbit polyclonal antibody against β-III tubulin (1:1000; Dako, Santa Clara, CA, USA) or mouse monoclonal antibody against PSD-95 (1:200; Abcam, Waltham, MA, USA) in blocking solution. After 3 washes in PBS, preparations were incubated for 20 min with Cy3-labeled anti-rabbit secondary antibody (1:50; Jackson Immunoresearch, West Groove, PA, USA) and FITC-labeled anti-mouse secondary antibody (1:50; Jackson Immunoresearch) in blocking solution. After three further washes in PBS, preparations were mounted with Prolong Gold Antifade (Invitrogen) and observed with a fluorescence microscope (Zeiss, Oberkochen, Germany).

### 2.3. RNA Isolation

Total RNA was extracted from neuronal cell cultures at different time points (days 4, 8, and 17, and at day 17, either 1 h or 12 h after NMDA stimulation) using Trizol (Invitrogen) according to the manufacturer’s protocol. RNA concentrations were determined using Nanodrop. For each extraction, 1 µg of RNA was treated with 1 unit of DNase (Invitrogen).

### 2.4. Quantitative Real-Time RT-PCR

Semi-quantification of E2 gene expression was performed by real-time quantitative RT-PCR. Reverse transcription reactions were performed at 42 °C for 45 min in a 25 µL reaction mixture containing 250 ng total RNA, 50 ng random hexamers, 25 mM dithiothreitol, 1 mM dNTP, 1× buffer, and 2U superscript II (Invitrogen). Reactions were followed by a 5 min incubation at 95 °C for 5 min. Primer sequences for the cDNA of the E2 genes, the *Ubiquitin* and *c-fos* genes, and two standardization genes *Gapdh* and *β-actin* are shown in Table S1. All primers were designed using Primer-Blast software. The PCR reaction solution consisted of 2.5 µL cDNA solution, 0.15 µM of each primer, and 5 µL Sybergreen qPCR supermix UDG (Invitrogen) in a final volume of 10 µL. The experiment was performed at Tm 60 °C using a LightCycler 480 system (Roche, Basel, Switzerland). For each primer set, PCR conditions were optimized in preliminary experiments to obtain a linear relationship between initial RNA concentrations (dilution series) and fluorescence levels (crossover point values), with PCR efficiencies close to 2. cDNA amplifications of the *Gapdh* and *β-actin* standardization genes were measured for each sample as an internal PCR control for sample loading and normalization. To quantify the relative level of gene expression for all target genes, we used the comparative crossover point (CP) method, including PCR amplification efficiency values (RealQuant and REST software; Roche). Reactions for each primer pair were performed three times on each RNA sample from three different neuronal cultures. Variation in E2 expression between two successive points in the culture was considered different when the ratio (Crossing Points, CPs) was ± 0.15.

### 2.5. Promoter Analysis

In silico analyses were performed on the promoters of all E2 genes expressed in hippocampal neurons. Jaspar software 4.0 was used to identify potential binding sites for transcription factors in a 1 Kb region 5′ to the first exon [42]. Only binding sites with a binding threshold above 7.5 (the threshold for the Creb binding site on the C-fos gene) were considered. Promoter regions were also analyzed using the EMBOSS CpGplot program v.6.2.0 (http://www.ebi.ac.uk/Tools/seqstats/emboss_cpgplot/, accessed on 1 October 2024).

## 3. Results

### 3.1. E2 Orthologs in Human and Mouse

**Figure 1:**
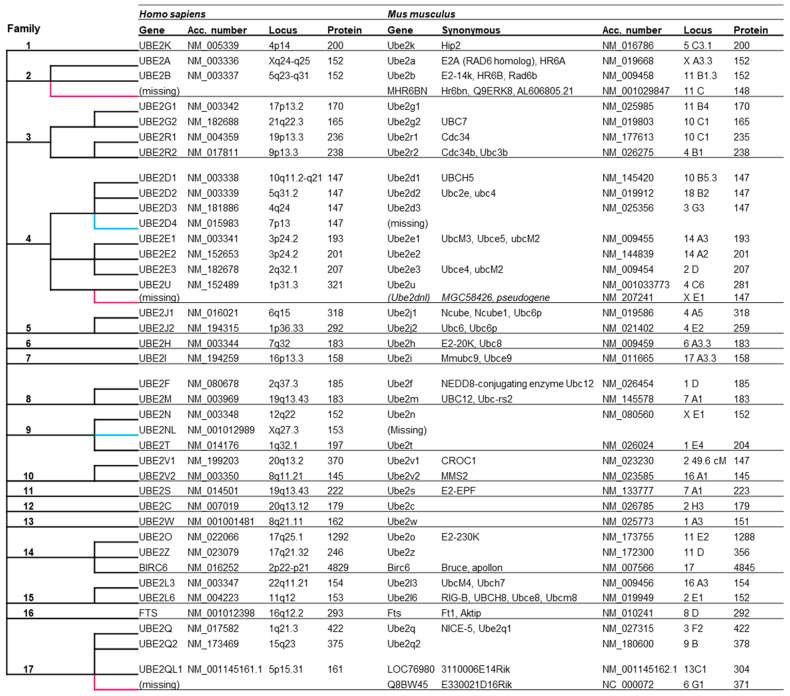
The classification of human E2s into 17 families (according to Michelle et al., 2009 [39]) and correspondence with mouse E2s. Acc. Number are the NM accession number of genes. Locus, chromosomal location of genes. (Missing) corresponds to the absence of orthologous genes in mice or humans.

A phylogenetic analysis led us to define 17 E2 families, with 37 E2 genes in the human genome [39] (Figure 1; Figure S1). We added the ubiquitin-conjugating enzyme E2Q family-like 1 encoded by the *UBE2QL1* gene at 5p15.31, which is not a pseudogene as previously suggested but participates in lysophagy [43]. We showed that the majority of these genes are present in the human genome, and encoding E2 enzymes are also present in the mouse genome (Figure 1). However, no ortholog was found in mice for the human genes *UBE2D4* in family 4 and *UBE2NL* in family 9. The Genebank database indicates the expression of the *Hr6bn* gene in mice, a family 2 gene absent in the human genome. *Hr6bn* encodes an enzyme with a classic E2 three-dimensional structure, i.e., four α helices, a PxxPP domain, an active cysteine, and a conserved tryptophan. We observed its expression in the frontal cortex of adult mice, but we did not detect the expression of the family 4 gene *Ube2u* (ortholog of human *UBE2U*) and the family 17 gene Q8bw45 (absent from the human genome) in adult mouse cortex; however, we did observe their expression in skeletal muscle. These two genes are also expressed in the testis (Symatalas database) and in the ovary of a two-day-pregnant adult female (Genbank database), respectively. Our studies in mouse cortex and skeletal muscle confirm the Genbank database’s conclusion that *Ube2dnl* may be a pseudogene. Taken together, these data indicate the presence of 38 E2 genes in the mouse genome, 36 of which are expressed in the brain.

### 3.2. Differential Expression of E2 Genes in Young Hippocampal Neurons (DIV4) in Cultures

**Figure 2:**
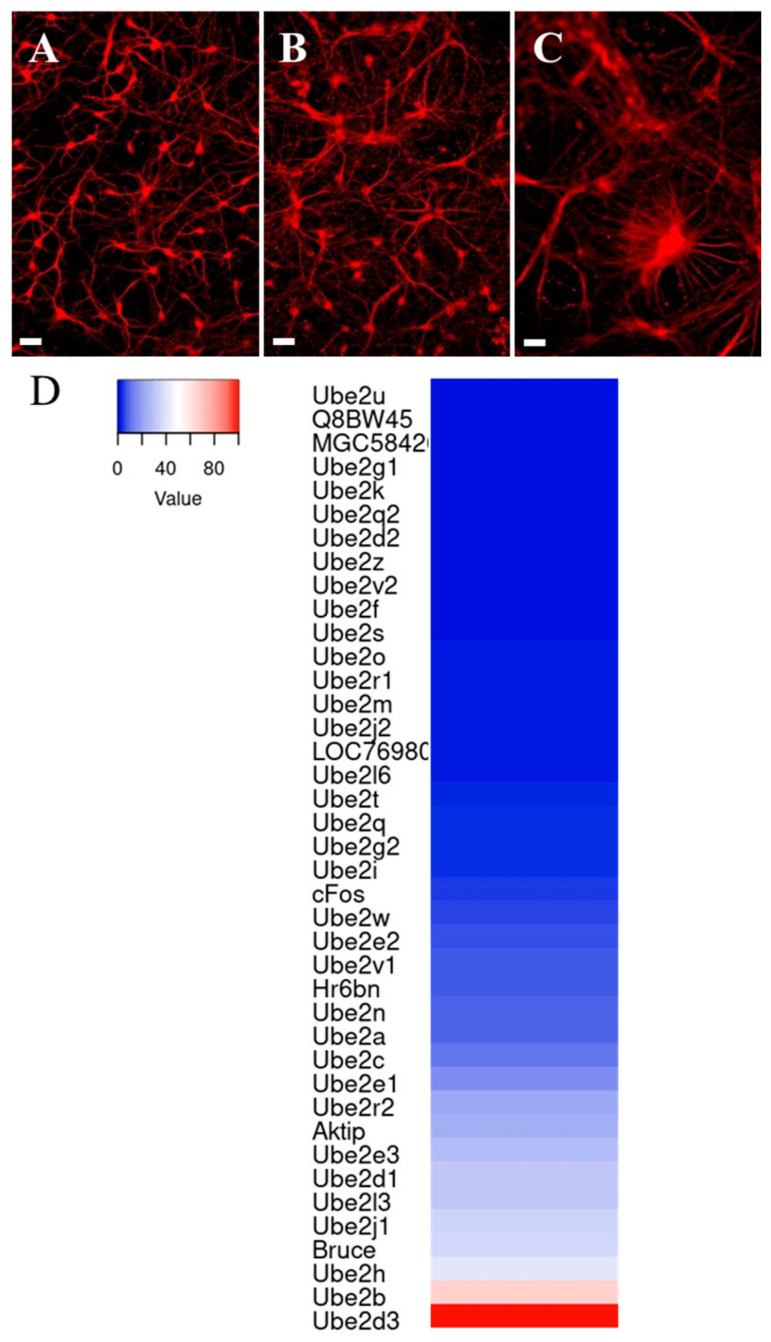
(**A**–**C**) A photograph of hippocampal neurons after immunocytochemistry against β-III tubulin at DIV4 (**A**), DIV8 (**B**), and DIV17 (**C**) (scale bar: 50 µm). (**D**) A heat map showing the relative expression levels of the E2 genes in hippocampal neurons at DIV4 (software heatmapper;) (www.heatmapper.ca; 1 October 2024).

We used embryonic cultures of hippocampal neurons to analyze E2 gene expression during neuronal differentiation. This cell culture model undergoes the same stereotyped sequence of developmental changes as in vivo [44]. After four days in vitro (DIV4), axonal and dendritic domains are already well defined, with a growing axon with branches and small dendrites (Figure 2A). We analyzed the expression of the 38 E2 genes in three independent batches of DIV4 hippocampal neuronal cultures. We first observed that 36 of the 38 E2 genes in the mouse genome were expressed in primary cultures of glutamatergic neurons from embryos. Only *Ube2u* and *Q8bw45* were not expressed according to our RT-qPCR analysis (Figure 2D).

We can subdivide the group of 36 E2 genes expressed in neurons from DIV4 into high, intermediate, and low expression levels. A total of 12 E2 genes from 9 families were high-expression-level genes. *Ube2d3* and *Ube2b* had the highest expression. Four genes were members of family 4, *Ube2d1*, *Ube2d3*, *Ube2e1*, *and Ube2e3*. Thirteen E2 genes from ten families showed intermediate expression levels. *Ube2i*, the unique gene encoding a SUMOylation E2 enzyme, and *Ube2l6*, encoding the main E2 enzyme for ISG15 conjugation, belong to this group. Finally, 11 genes from 8 families were weakly expressed. The two E2 neddylation genes, Ube2m and Ube2f, belong to this group. Taken together, these results indicate that the majority of E2 genes in the Ub/Ub-like pathways are expressed in young neuronal cells but with differences in intensity.

### 3.3. Differential Expression of E2 Genes During Neuronal Differentiation and Maturation

In developing hippocampal neuronal cultures, dendritic processes began to elongate rapidly from DIV5 [44]. By DIV8, neurons showed a single long axon and several long, still-growing dendrites (Figure 2B). Between DIV8 and 17, neurons underwent what is known as the maturation process, with neurite and branch elongation and contact, and synaptogenesis [45]. At DIV17, a dense network of hippocampal neurons with dendritic spine-like structures was visible [46] (Figure 2C).

**Figure 3:**
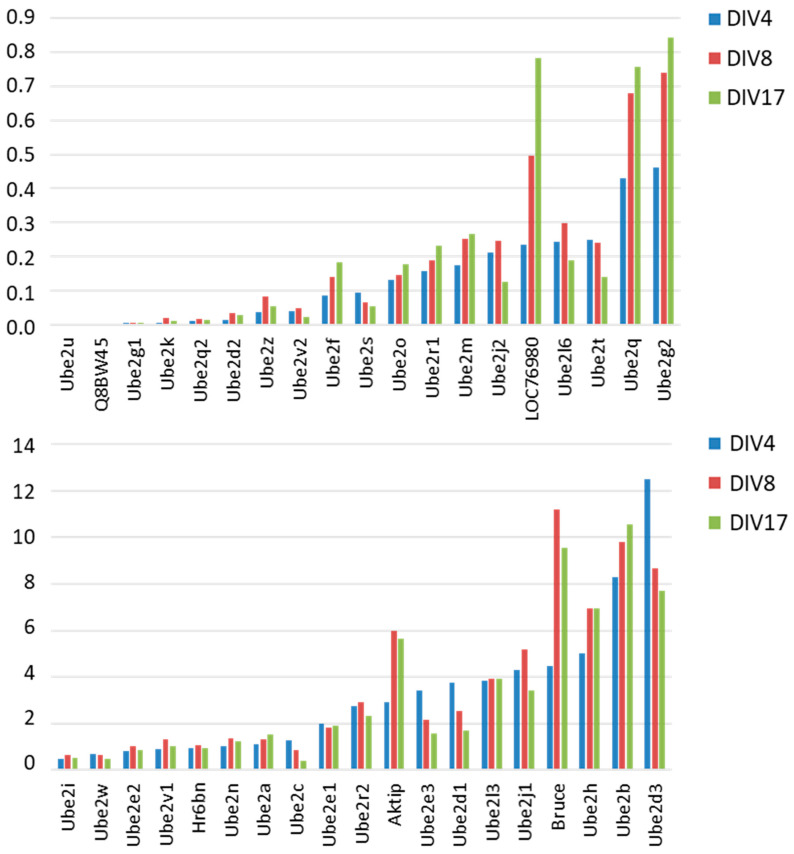
Variation in E2 gene expression between periods DIV4, DIV8, and 17 (expression ratios). Genes were classified according to their expression level at DIV4.

We analyzed the expression profiles of the 36 E2 genes during the axonal branching and dendritogenesis stage from DIV4 to DIV8 and the maturation stage from DIV8 to DIV17 (Figure 3). Fourteen E2 genes showed increased expression during the differentiation and maturation periods, in particular *LOC76980* (orthologous to *UBE2QL1*), *Bruce* (orthologous to *BIRC6*), *AKTIP*, *Ube2q*, and *Ube2g2*. Interestingly, these genes are concentrated in certain E2 families (2, 6, 8, 14, 16, and 17). *Ube2b*, a member of family 2, is required for neurite growth and is the paralog of *Ube2a*, the murine orthologue of *UBE2A* mutated in X-linked intellectual disability [47]. Conversely, other families contain several genes, including *Ube2e3*, *Ube2d1*, or *Ube2d3*, which show a decrease in expression during both periods (families 4, 9, 11, and 12).

Different expression profiles were found for other genes when comparing differentiation and maturation stages. A biphasic profile was observed for family 5 genes, *Ube2j1* and *Ube2j2*, whose expression increased during differentiation and then decreased during maturation. Other genes showed little change in expression during these two periods. This is the case for *Ube2n*, homologous to the Drosophila growth cone guidance gene *bendless* [48]. Taken together, these results show significant changes in the regulation of the expression of a majority of E2 genes during neuronal differentiation and neuronal maturation.

### 3.4. Variation in E2 Gene Expression During Short and Late-Responses to NMDA

**Figure 4:**
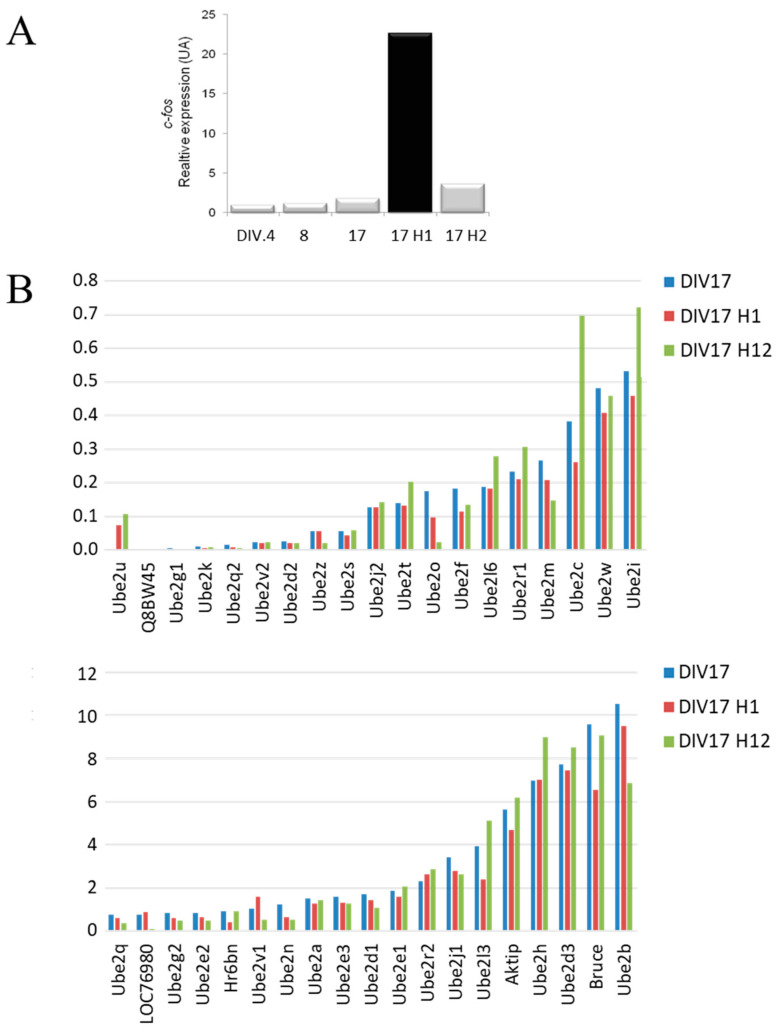
(**A)** Variation of C-fos gene expression during neuronal differentiation and after NMDA stimulation. (**B**) Variation in E2 gene expression before (DIV17) and after NMDA stimulation (1 h and 12 h) (expression ratios). Genes were classified according to their expression level before stimulation at DIV17.

Numerous studies support an important role for Ub/Ub-like pathways in synaptic function and plasticity in the brain [4,6,7]. We analyzed the expression of all E2 genes in mature hippocampal glutamatergic neurons (DIV17) after stimulation with the glutamate receptor agonist N-methyl-D-aspartate (NMDA). Neuronal stimulation was confirmed by a significant increase in the expression of the early response gene *C-fos* 1h after the addition of NMDA (Figure 4A). Several E2 genes showed no variation after stimulation at 1h or 12h. Others show a decrease in expression at both times after stimulation, such as *Ube2j1*, *Ube2n*, *Ube2o*, and one of the Nedd8 E2 genes, *Ube2m*. The other E2 genes showed different regulation at the early (1h) and late (12h) periods after NMDA stimulation. For example, *Ube2v1*, whose function in neurons is still unknown, was up-regulated only during the early post-stimulation period. Several genes showed increased expression only during the late period. These include *Ube2l6*, which regulates the synaptic vesicle protein synaptophysin [49], and *Ube2l3*, which is involved in the degradation of caV2.2 channels in neurons [50]. This was also the case for *Ube2c*, *Ube2h*, and the SUMOylation E2, *Ube2i*. Overall, our data indicate that many E2 genes are subject to activity-dependent regulation in neurons.

### 3.5. Analysis of the Regulation of Expression of Genes Encoding E2s

**Figure 5:**
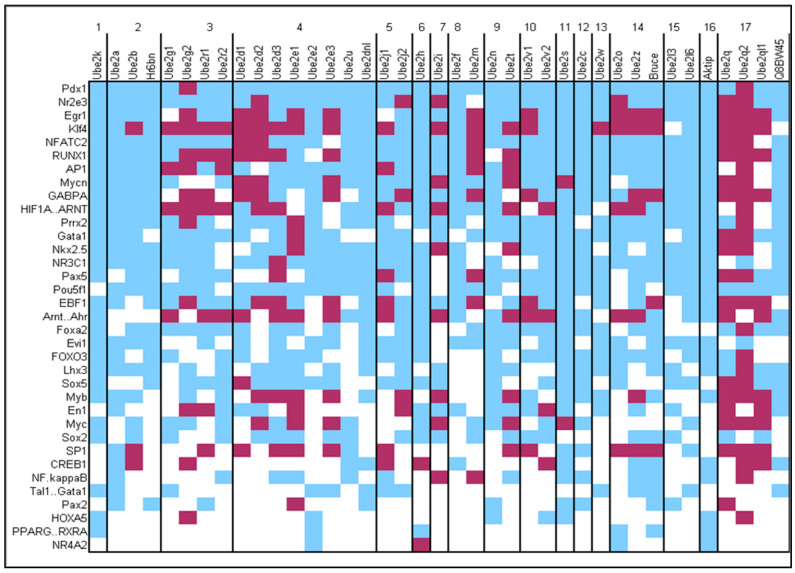
A schematic representation indicating the presence (color) or absence (white) of potential transcription factor binding sites in the promoter sequence of the E2 genes classified by families. The sites located in CpG islands are in dark red, and the others are in blue.

To better understand the regulation of genes encoding E2s during neuronal differentiation, during maturation, and after stimulation, we searched for the presence of putative transcription factor (TF) binding sites (BDs) in the promoter of all genes encoding E2s using Jaspar core database. BDs for 53 TFs (out of 125 TFs listed in the database), 34 of which are known to be expressed in neurons, were found in the promoter sequences of the 38 E2 genes (Figure 5). BDs for the Pdx1 homeodomain TF were present in all 38 E2 genes. Moreover, 13 TFs had BDs present in at least one gene from all 17 E2 families. Using the Emboss CpGplot program, we observed that some of these BDs were very often located in CpG islands, such as BDs for Gabpa and Klf4, whose expression is triggered by neuronal activity (Figure 5) [51]. We also analyzed the data according to E2 families. Similar patterns of BDs were present within families, supporting the phylogenetic classification of E2 proteins based on protein sequences [39] (Figure 5). For example, families 1, 2, 11, 12, 15, and 16 contained few or no BDs located within CpG islands, compared to other E2 families. Conversely, family 17 contained a majority of BDs located within CpG islands. This family appeared late in evolution.

We then compared BD patterns for TFs with expression profiles within E2 families (Figure 5). The two neddylation genes *Ube2f* and *Ube2m* (family 8) showed differences in BDs, such as a frequent localization of BDs within CpG islands in *Ube2m* but not in *Ube2f* and for *Ube2f* but not *Ube2m*, the presence of a putative BD for CREB1, a TF involved in the signalling pathway after NMDA receptor activation on neurons. These observations confirm the different expression profiles observed for these two genes after NMDA stimulation, i.e., stability for *Ube2f* and down-regulation for *Ube2m*. A similar observation was obtained for *Ube2t* and *Ube2n* of family 9. We observed that 13 of the 36 E2 genes expressed in neurons contained putative BDs for CREB1. The presence of these BDs was heterogeneous within families, but it is interesting to note that all 7 genes with CREB1 BDs located within CpG islands, except *Ube2h and Ube2v2*, were down-regulated 12h after NMDA stimulation, suggesting a possible involvement of DNA methylation in the regulatory mechanisms. *Ube2b* of family 2 was one of these genes. In the same family 2, *Ube2a*, whose human ortholog is mutated in intellectual disability, contained a putative CREB1 BD outside a CpG island. Altogether, these data revealed similarities in the promoter regions of E2 genes within families, confirming phylogenetic data on ubiquitin-conjugating enzymes and providing insights into the regulation of their expression during development and neuronal activity.

## 4. Discussion

The ubiquitination process plays an essential role in various stages of CNS development and maturation, including neurite growth and synaptogenesis [14,30]. The action of E2s in these Ub/Ub-like pathways is central, and some Ub-like pathways involve only a single E2, making them essential. Several mechanisms regulate E2 activity, namely non-covalent modulations, covalent modulations such as ubiquitination or SUMOylation of certain E2s themselves, and modulation through the control of transcription of E2 encoding genes [52]. Here, we showed that the majority (36 out of 38) of E2 genes are expressed in hippocampal neurons and that this expression is dynamic during neuronal development and maturation. A majority of E2s are up-regulated during the period of differentiation corresponding to neurite growth; then, most genes show a down-regulation of expression during maturation (branch elongation) and synaptogenesis. This dynamic expression of E2s, which we observed in cultured neurons, is in line with the results of studies in humans at a more global scale of the developing brain [53]. Furthermore, our results complement several previous studies. For example, it has been shown that the down-regulation of *Ube2b* mRNA in PC12 cells leads to an NGF-induced reduction in neurite length. This result stems from the observation that *Ube2b* expression increases during the elongation stage of neurites and branches in neuronal cultures. Overall, this work supports the idea to perform functional studies on several genes encoding E2s, such as *Ube2d3*, for example, whose expression varies during neuronal development and which has been characterized as a neuronal protective factor under various neurodegenerative stresses. Another example is *Ube2l3*, which is the E2 of the ligase enzyme ARIH2 very recently identified mutated in a patient with autism spectrum disorder and intellectual disability [54,55]. ARIH2 is involved in the regulation of Hedghehog signaling, a pathway crucial for neurogenesis and neural patterning during the development of the CNS and whose genes continue to be expressed in differentiated neurons [56,57].

Several studies have also shown the involvement of Ub and Ub-like pathways in the functioning and regulation of synaptic activity [58,59]; These pathways are, among other things, directly involved in the control of synaptic connections during memory and learning processes, among other things, by regulating the trafficking or internalization of receptors at the synapse as well as their degradation [13,60]. The activation of glutamate receptors on mature neurons is known to produce short- and long-term synaptic modifications through the activation of molecular pathways and changes in gene expression [61,62,63]. Interestingly, the majority of E2 genes are subject to an activity-dependent up- or down-regulation of expression, supporting their involvement in synaptic functions. The deregulation of the expression of these genes could be directly or indirectly involved in NDDs. For example, the conditional knockout of *Ube2n* in mice results in impaired walking, spontaneous locomotion, and exploration [64].

Several E2s show variations in expression in Alzheimer’s disease, Parkinson’s disease, and amyotrophic lateral sclerosis (ALS). For example, age-associated reduction in *UBE2O* gene expression promotes neuronal death in Alzheimer’s disease. Interestingly, we show that NMDA-mediated synaptic activity reduces the expression of this *Ube2o* gene. There may therefore be a link between the variation in *Ube2o* expression and the fact that NMDA receptor activation is involved in Alzheimer’s disease [65,66]. In Parkinson’s disease, a variation in the expression of the E2 enzyme, UBE2L3 (UbcH7), has been observed. This enzyme constitutes the optimal E2 for the E3 Parkin involved in Parkinson’s disease [67,68]. Another example in Parkinson’s disease is the E2 UBE2K, whose expression is decreased in the substantia nigra of patients [69]. UBE2K, also known as HIP2 (HTT Interaction Protein 2), interacts with wild-type and mutated forms of Huntingtin protein. Thus it would be interesting to study its expression in striatum tissue from Huntington’s disease patients [70]. This same E2 Ube2k is up-regulated in the spinal cord of SOD1-G93A mice, a model of amyotrophic lateral sclerosis (ALS), another NDs. It protects cells against toxicity induced by the mutation of the SOD1 gene [71]. Another interesting E2 example in the pathophysiology of ALS is represented by the three members of the UBE2E family, which may promote the ubiquitination of TDP-43, a protein present in aggregates in degenerating neurons in the majority of patients. [72,73]. These three E2 enzymes are expressed during neuronal stimulation. Interestingly cortical hyperexcitability is an important mechanism involved in the pathophysiology of ALS and is linked to TDP-43 mislocalization observed in ALS [74,75]. Moreover, these three genes encoding UBE2E enzymes contain binding sites for the same TFs expressed in neurons, such as AP1. The DNA-binding activity of AP1 is known to increase after the addition of glutamate to cultured neurons [76].

To conclude, our results show that the regulation of E2s expression is dynamic during development, neuronal maturation, and synaptic stimulation in mouse hippocampal neuron culture. This culture is a widely used model for studying the pathophysiology of many NDDs and NDs. It is also used for the pre-clinical testing of potential therapeutics. We have identified a series of E2-encoding genes, which now need to be extensively studied at these three stages, both physiologically and under pathophysiological conditions involving genetic or environmental causes. Knowledge of the potential binding sites of TFs present in E2s promoters is a valuable source of information for these studies. These findings, combined with the fact that we have more information on the structure of E2s, could lead to new tools for the study of Ub/Ub-like pathways and possibly to new drugs for the treatment of NDD or ND. Based on the results of this study, it will now be possible for all teams producing other primary neuron cultures (dopaminergic, GABAergic, for example) or various IPSC-derived neuron cultures to target studies on the expression of certain E2 genes according to their neuron type of interest and cell function of interest, i.e., differentiation, maturation, or synaptic activity.

## Data Availability

Data is contained within the article or Supplementary Material.

## Supplementary Materials

The following supporting information can be downloaded at: https://www.mdpi.com/article/10.3390/genes15111381/s1, Figure S1: Schematic representation of the 23 pairs of human chromosomes with the location of the 37 E2 genes.; Table S1: Primers used for RT-qPCR experiments.
