# Supplementary material for: Dynamic Expression of Genes Encoding Ubiquitin Conjugating Enzymes (E2s) During Neuronal Differentiation and Maturation: Implications for Neurodevelopmental Disorders and Neurodegenerative Diseases
Source: Genes (Basel). 2024 Oct 26;15(11):1381. doi: 10.3390/genes15111381 (PMC11593721; doi:10.3390/genes15111381)
Supplement: Supplementary file 1 [file genes-15-01381-s001.zip › genes-3262892-supplementary.pdf]

Supplementary Table S1: Primers used for RT-qPCR experiments.

GENES	Forward	Reward
**Aktip**	CCATCTTACCGCTCTGCATT	GGACAGTCGCCATCAGGATA
**Bruce**	TCTTACCTCTTGCCCAGTGC	TGCCCACCTATAGCCAACAT
**Hr6bn**	AGCGATTGCAAGAGGACCTA	TGTCCCGTCTGGTCCAAATA
**Ube2ql1**	CTGGTCCAGCGCCTACAC	AGGTTGCTTCAGCTTCCTTG
**Ube2dnl**	TAGTTGCAAGCTGTGAGGGA	GCTGTTATGCAGTTGCTGGA
**Q8BW45**	GAGGAAGGATGTCGTCAGGT	CTGTAGGTCATCCAGGTGCC
**Ube2a**	TGAAGAGTATCCTAATAAGCCACCT	GGACTCCAACGGTTCTGAAG
**Ube2b**	GAATCGATGGAGTCCCACAT	GTTGGCTGGACTGTTTGGAT
**Ube2c**	TGATGTCAGGACTATCTTGCTCTC	TGGGGTTTTTCCAGAGTTCC
**Ube2d1**	TGTTCCACTGGCAAGCAA	AGTCTGTCGGAAAGTGGACAG
**Ube2d2**	CTTCAAACCGCCTAAGGTTG	TGGAGACCACTGTGACCGTA
**Ube2d3**	TTAGTGATTTGGCCCGTGAC	GGTCCCATAATTGTGGCTTG
**Ube2e1**	AGGAGCTGGCAGACATCACT	CCTTCATACACAGACCCTGGA
**Ube2e2**	GAGCTTGCAGAAATCACGC	TCATAGACAGATCCTGGGGG
**Ube2e3**	AACCCTTGATCCTCCTCCTAA	CACCTTCATATACAGAACCTGGTG
**Ube2f**	AAATGAGTAACATCAGGCCACA	TGTGGGGAAACCTGAATGAT
**Ube2g1**	CCGGCCTCCTAAAATGAAAT	CCCCAGGCTCATGAAGAATA
**Ube2g2**	GGTTATGAGAGCAGTGCCG	CACTCTCATCGTTGGGCTCT
**Ube2h**	GGACTCAATGAATTTGTTGTCAA	AGGGTATTTATCAGGAAGGTCCA
**Ube2i**	GAGGCTTGTTCAAGCTACGG	CTGTGCCAGAAGGATACACG
**Ube2I6**	ATCTACCACCCCAACGTCAG	GAGGGCCTCCAAGACTTGAT
**Ube2j1**	AGTACCCCATGAAACCACCA	GCCAGGTTTCAGGATGATGT
**Ube2j2**	GCAACACAAAGGTTGAAGCA	AGGGCCTCGGACAACATAAT
**Ube2k**	AAATACCAGAAACATATCCATTTAACC	TCCAAACAAATAGCCCCTGT
**Ube2l3**	TTGGCAAGGGCTTATTGTTC	TGTGATCTTGGGTGGTTTGA
**Ube2m**	CAGCTTTAAGGTGGGACAGG	AGGATGTTGAGGCAGACGTT
**Ube2n**	GCTCCTAATCCAGATGATCCA	AGTCCATGCTCTCGCTGTTT
**Ube2O**	CCCACCTTCCATCATCACTC	CTACCTTGGCTGGGAAGACA
**Ube2q**	TACAACCTCCCTCAGCATCC	CTCAGGCATCTCTTCATCTTCA
**Ube2q2**	TGGGAAAAAGTCAGAGGATGA	ACCCAGACACTGCACCATTT
**Ube2r1**	AAGTTCCCCATCGACTACCC	TGGAGAATGGAGATGCACAC
**Ube2r2**	ACCCTCTGAAAGGTGGAACC	TTGGCTGGAGAAAAGGTGTT
**Ube2s**	CCCAATGAGGAGGATCTCAC	TCCTTCCCCAGTAGGAGCTT
**Ube2t**	AACCTCCACAGGTCCGATTT	TGTTGAGGGATGGTCTCCA
**Ube2u**	TGAGATTGAAGGTCTTCGCA	TTTCACATTTGGAGGAACAGAA
**Ube2v1**	CACCCCCGTCTGTAAGATTC	TTGATGCTGTGGGAGTTCTG
**Ube2v2**	CAGCAACCTTCTACTCATGTTAATG	GTCCAGGTCATGTTTACCAGC
**Ube2w**	CGACCACCAGATAATTCCTTTT	CGGCATCAACAAGTGTCATC
**Ube2z**	ATCAGGGTTGCAGTCTGTGA	CTTTGCAGGCCACCTCATA
**Gapdh**	CTGCACCACCAACTGCTTAG	GTCTTCTGGGTGGCAGTGAT
**B Actin**	GACAGGATGCAGAAGGAGATTACT	ATCTGCTGGAAGGTGGACAG
**c Fos**	TCACCCTGCCCCTTCTCA	CTGATGCTCTTGACTGGCTCC

Supplementary Figure S1: Schematic representation of the 23 pairs of human chromosomes with the location of the 37 E2 genes.

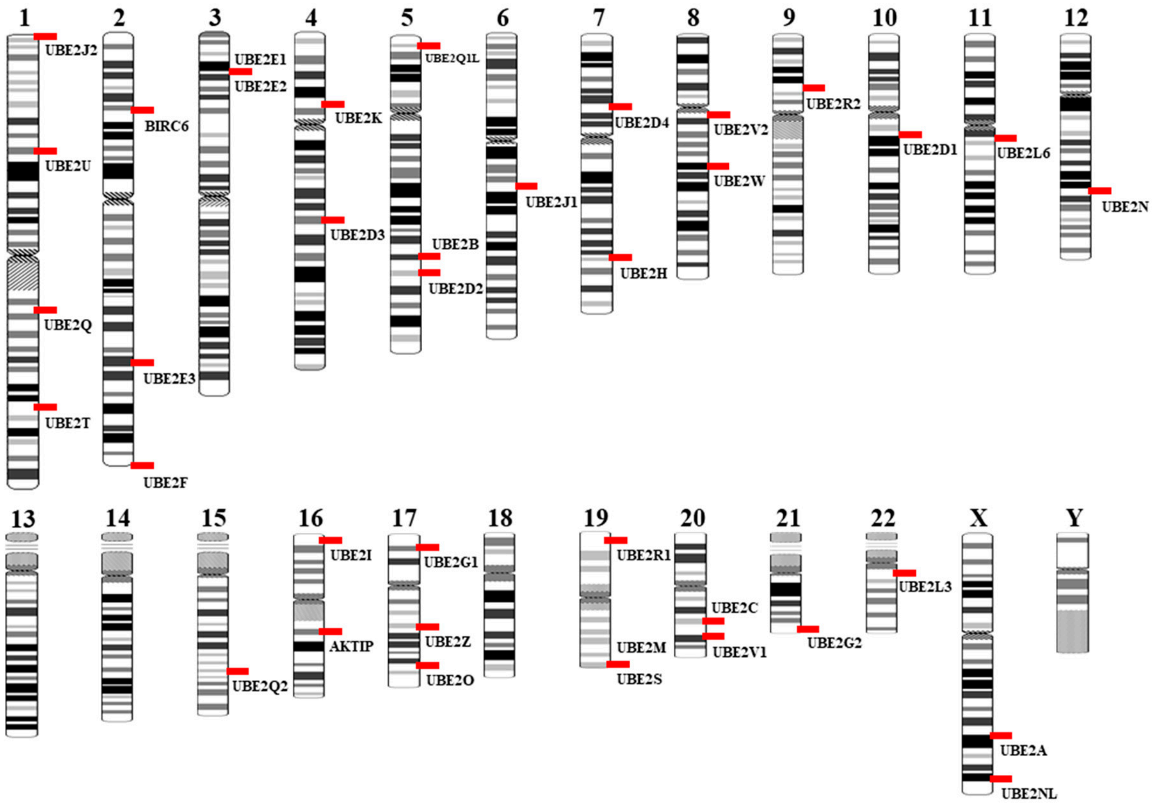
1
2
3
4
5
6
7
8
9
10
11
12
UBE2J2
UBE2U
UBE2Q
UBE2T
BIRC6
UBE2E3
UBE2F
UBE2E1
UBE2E2
UBE2K
UBE2D3
UBE2Q1L
UBE2B
UBE2D2
UBE2J1
UBE2D4
UBE2H
UBE2V2
UBE2W
UBE2R2
UBE2D1
UBE2L6
UBE2N
13
14
15
16
17
18
19
20
21
22
X
Y
UBE2Q2
UBE2I
AKTIP
UBE2G1
UBE2Z
UBE2O
UBE2R1
UBE2M
UBE2S
UBE2C
UBE2V1
UBE2G2
UBE2L3
UBE2A
UBE2NL
